# Chemogenetic silencing of the subiculum blocks acute chronic temporal lobe epilepsy

**DOI:** 10.1186/s13041-024-01164-9

**Published:** 2024-11-29

**Authors:** Jianbang Lin, Jing Liu, Qi Zhang, Taian Liu, Zexuan Hong, Yi Lu, Cheng Zhong, Zhonghua Lu, Yuantao Li, Yu Hu

**Affiliations:** 1 Shenzhen-Hong Kong Institute of Brain Science, Shenzhen Institute of Advanced Technology, Chinese Academy of Sciences, Shenzhen, 518055 China; 2 University of Chinese Academy of Sciences, Beijing, 100049 China; 3 Department of Anesthesiology, Affiliated Shenzhen Maternity & Child Healthcare Hospital, Southern Medical University, Shenzhen, 518027 China; 4 Department of Medicinal Chemistry and Natural Medicine Chemistry, College of Pharmacy, Harbin Medical University, Harbin, 150081 China; 5 The Key Laboratory of Biomedical Imaging Science and System, Chinese Academy of Sciences, Shenzhen, 518055 China; 6 Biomedical Research Institute, Hubei University of Medicine, Shiyan, 442000 China

**Keywords:** Temporal lobe epilepsy, Kainic acid, Generalized tonic–clonic seizures, Subiculum, Chemogenetic

## Abstract

Temporal lobe epilepsy (TLE) is the most common form of medically-intractable epilepsy. Subicular hyperexcitability is frequently observed with TLE, presumably caused by impaired inhibition of local excitatory neurons. Here, we evaluated the effectiveness of silencing subicular pyramidal neurons to treat a rodent model of TLE. First, we generated a chronic TLE mouse model via initial intrahippocampal kainic acid (IHKA) injection. In the chronic state after first IHKA injection, behavioral seizures and histological abnormalities were reliably observed. We then injected an adeno-associated viral (AAV) vector carrying an inhibitory chemogenetic element, hM4D_i_, directly into the subiculum. Eight weeks after the first IHKA injection, acute seizures were induced by giving a second dose of kainic acid (KA), which mimicked generalized tonic–clonic seizures. Herein, precise control over generalized tonic–clonic seizure onset was achieved via this two-step process. We found that chemogenetic suppression of subicular pyramidal neurons had a robust anti-epileptogenesis effect in this acute-chronic model of TLE. These data confirm a crucial role of the subiculum in the propagation of hippocampal seizures and highlight the potential for using subicular chemogenetic manipulation to treat generalized tonic–clonic seizures.

## Introduction

Temporal lobe epilepsy (TLE) is the most common cause of intractable seizures, and 20–30% of TLE patients fail to become seizure-free despite pharmacotherapy [1, 2]. Although the etiology of TLE remains largely elusive, recent advances in our understanding of implicated brain structures and neural circuits [3] provide a basis on which more effective and precise intervention strategies might be developed [4]. Pathological changes in TLE brains are mainly restricted to temporal lobe regions, especially the hippocampus. Both TLE patients and animal models exhibit heightened excitability in the subiculum [5, 6], a key hippocampal output region that connects to areas such as the retrosplenial cortex, the nucleus accumbens, and the thalamus. Activation of GABAergic neurons in the subiculum immediately following status epilepticus (SE) can restrain the spread of focal ictal activity from the onset zone, and thereby prevent generalization [7]. These observations all indicate that the subiculum is a promising target for the prevention of hippocampal seizure spread and generalization [8, 9]. Moreover, a recent study reported that a hyperactive subiculum is largely responsible for pharmaco-resistance in TLE [10]. Given this evidence, investigation of the effects of interventions to reduce neuronal activity in the subiculum following TLE seizures is warranted.

One strategy involves the use of designer receptors exclusively activated by designer drugs (DREADDs), engineered G protein-coupled receptors that respond selectively to inert synthetic ligands. These allow for remote and regulable control of neuronal activity. Therapeutical effects of such DREADDs on focal onset seizures have been well documented in several studies. For example, Kätzel and colleagues report the attenuation of chemoconvulsant-induced focal epilepsy in the rat motor cortex using hM4D_i_ delivered by adeno-associated virus (AAV) [11]; systemic administration of the hM4D_i_ agonist clozapine-N-oxide (CNO) led to the suppression focal seizures. In addition, it has been shown that DREADD-mediated chemogenetic silencing of the midline thalamus can suppress limbic seizures [12] and suppression of excitatory hippocampal neurons mediating seizure suppression in chronic TLE models [13, 14]. However, research using DREADDs to treat generalized seizures is lacking.

Here, we sought to evaluate the efficacy of chemogenetic silencing of the subiculum in suppressing generalized seizures evoked from the hippocampus in our acute chronic TLE mouse model. First, we developed a chronic TLE mouse model using intrahippocampally kainic acid (IHKA) injections. Once established, we consistently observed both behavioral seizures and histopathological abnormalities. We then manipulated neuronal activity within the hippocampal circuitry, specifically, at the level of the subiculum. To achieve this, we administered an AAV vector encoding an inhibitory chemogenetic receptor (hM4D_i_) directly into the subiculum and allowed 3 weeks for viral expression. Subsequent activation of hM4D_i_ following CNO administration led to a clear inhibition of kainic acid (KA) kindling-induced seizures. Together, this demonstrates the feasibility of using chemogenetic silencing of the subiculum to curb the spread and generalization of TLE. This finding not only offers a novel perspective on the pathophysiology of the disease but also paves the way for targeted therapeutic strategies.

## Results

### Hippocampal subregions are morphologically altered in a KA-induced chronic TLE mouse model

**Fig. 1:**
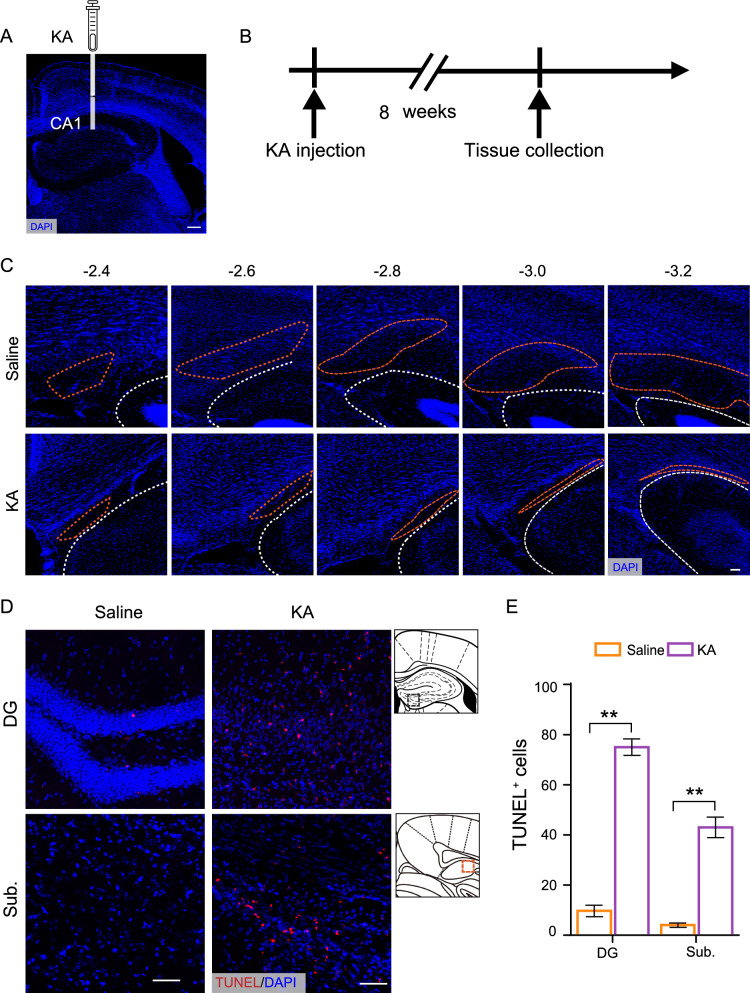
Changes in hippocampal apoptosis and morphology in a chronic kainic acid-induced TLE mouse model. **A** Schematic showing an intrahippocampal kainic acid injection (IHKA) in a mouse. Scale bar, 250 µm. **B** Experimental timeline showing the IHKA microinjection model of chronic TLE. **C** Representative DAPI-stained coronal sections showing hippocampus sections between − 2.4 mm and − 3.2 mm from bregma. The top row represents sections from the KA group, while the bottom row represents sections from the saline group. The white dashed areas represent the dentate gyrus (DG), and the orange dashed areas represent the subiculum (Sub.). Scale bar, 50 $\upmu$m. **D** Representative images of TUNEL-positive cells (red) in the DG and Sub. The schematic diagrams on the top right illustrate the selected regions. Scale bar, 100 µm. **E** Quantitative cell counts of TUNEL-positive cells across the DG and Sub. n = 3 mice per group, Data presented as mean ± SEM, two-tailed unpaired *t* test; *P < 0.05, **P < 0.01, n.s, not significant

To establish a chronic TLE model, KA was administered intrahippocampally at a dose of 10 µg/kg. Following an eight-week period, brain tissues were harvested for both histological and molecular analyses (see Fig. 1A, B for schematic diagram and timeline). DAPI staining revealed significant structural distortion of the dentate gyrus (DG) and an altered distribution of granule cells in KA-induced mice compared to saline mice. These changes were observed across multiple anterior–posterior levels (AP − 2.4, − 2.6, − 2.8, − 3.0 and − 3.2 mm; white dashed areas in Fig. 1C). A close examination of the DG revealed morphology comparable to that observed in the saline group, albeit with a noticeably sparser distribution of granule cells within the DG layers (Fig. 1D). Interestingly, the morphology of the subiculum revealed a reduction in both volume and complexity, indicating substantial alterations in this region (orange dashed areas in Fig. 1C). These alterations were characterized by disrupted organization and decreased neuronal density within the subiculum, potentially implicating this structure in the pathophysiology of TLE.

To quantify the extent of cellular damage and apoptosis, TUNEL (Terminal deoxynucleotidyl transferase dUTP Nick End Labeling) assays were performed, revealing a marked increase in TUNEL-positive cells in both the ipsilateral DG and subiculum following KA induction (DG, 74.3 ± 4.6; subiculum, 42.3 ± 5.8, Fig. 1E), compared to saline controls (DG, 9.0 ± 3.3, *P* < 0.01; subiculum 3.3 ± 1.2,* P* < 0.01, Fig. 1E). These findings collectively highlight the profound effect of KA administration on hippocampal subregions, particularly the DG and subiculum, in the context of chronic TLE modeling, and offer insights into potential targets for therapeutic intervention.

### Projections to the subiculum are degraded in the chronic TLE mouse model

**Fig. 2:**
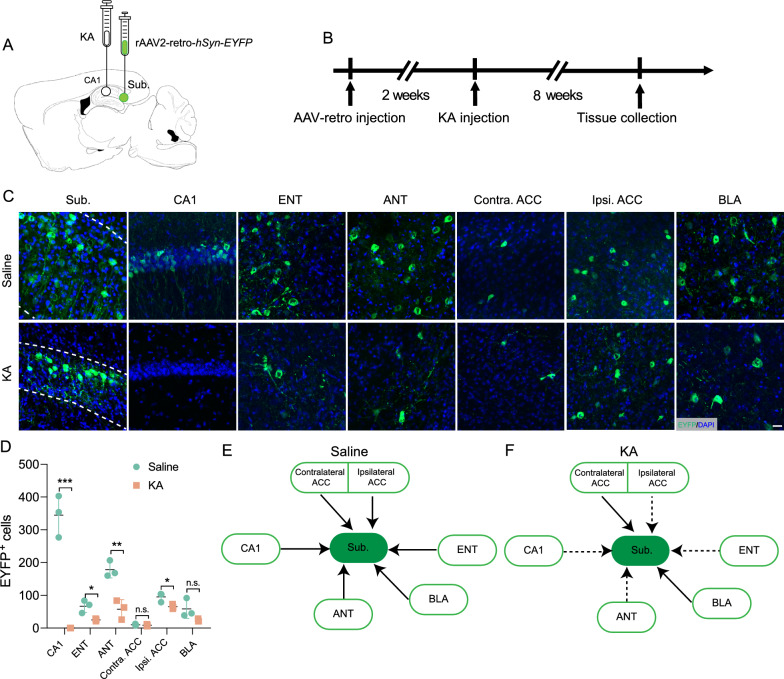
Disrupted circuitry in the subiculum of kainic-acid-induced TLE mouse brains. **A**, **B** Schematic representation (**A**) and experimental timeline (**B**) showing the strategy of retrograde tracing targeting the Sub. **C** Coronal mouse brain sections illustrating EYFP fluorescence marking the injection site in the Subiculum (Sub.) and retrogradely traced neurons in the CA1, entorhinal cortex (ENT), anterior thalamic nuclei (ANT), contralateral and ipsilateral anterior cingulate cortex (ACC), and the basolateral amygdala (BLA). Scale bar, 20 µm. **D** Quantitative cell counts of EYFP-positive neurons across the observed brain regions. n = 3 mice per group, Data presented as mean ± SEM, two-tailed unpaired *t*-test; *P < 0.05, **P < 0.01, ***P < 0.001, n.s., not significant. **E**, **F** Diagrammatic representation showing the effects of KA-induced remodeling of neural circuit inputs to the Sub., where dashed lines indicate reduced input post-KA injection (**F**)

Given the altered morphology and elevated apoptosis in the subiculum, we next tested whether neural projections to subiculum were remodeled in chronic TLE mice. We injected rAAV2-retro-*hSyn-EYFP* into the subiculum of chronic TLE mice (Fig. 2A, B), and following histological analysis, detected obvious EYFP signals around the injection sites, including subicular neurons and the projecting fibers, in addition to upstream brain regions (Fig. 2C). Subsequently, the overall input patterns of subicular neurons in chronic TLE mice were compared to a saline group. In the saline group, we observed dense projections to subicular neurons from several brain regions: the CA1, the entorhinal cortex (ENT), the anterior thalamic nuclei (ANT), the contralateral and ipsilateral anterior cingulate cortex (ACC), and the basolateral amygdala (BLA) (CA1, 344.7 ± 51.9; ENT, 67.0 ± 15.8; ANT, 178.3 ± 20.5; contra ACC, 10.3 ± 2.1; ips ACC, 95.3 ± 11.6; BLA, 58.7 ± 23.7; Fig. 2C, D).There was a similar pattern of projections received by subicular neurons in both the saline and KA group. However, the signal in both the subiculum and the mapped upstream input areas was dramatically lower in the KA group, including the CA1, ENT, ANT, contralateral and ipsilateral ACC (CA1, 0,* P* < 0.001; ENT, 25.3 ± 3.3,* P* < 0.05; ANT, 57.7 ± 24.0,* P* < 0.01; contra. ACC 9.0 ± 2.4; n.s; ips. ACC 65.0 $\pm$ 5.9,* P* < 0.05; BLA, 23.7 $\pm$ 3.8, n.s; Fig. 2C, D). Based on the findings of retrograde tracing experiments, we delineated the potential input circuitry of the subiculum and characterized how these are altered in a chronic mouse model of TLE (Fig. 2E, F). This suggests that the subiculum is a potential pivotal therapeutic target for TLE, essential for both intervention and understanding of the disease pathology.

### Characterization of the KA-induced acute chronic TLE mouse model

**Fig. 3:**
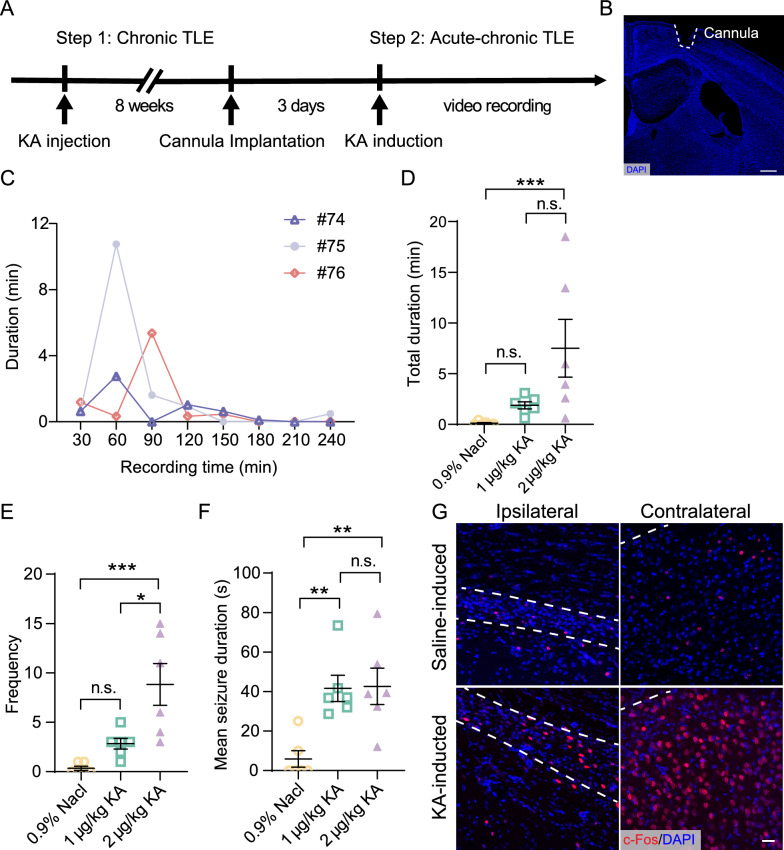
Characterization of intrahippocampal kainic acid microinjections as a model for chronic TLE. **A** Experimental timeline for the characterization of the acute-chronic TLE model. **B** Representative images showing the cannula position above the hippocampus. Scale bar, 250 µm. **C** Line plot of seizure intensity over time for three individual mice (#74, #75, #76), showing the seizure duration in minutes for each 30-min interval following KA administration. **D**–**F** Seizure characteristics in acute KA-induced groups (1 µg/kg and 2 µg/kg) and saline-induced groups (0.9% NaCl). Quantification of total duration (**D**) seizure frequency (**E**) and mean seizure duration (**F**) within 2 h following administration. Each symbol represents an individual animal, and color coding indicates KA or saline. n = 6 mice per group, data are represented as mean ± SEM, one-way ANOVA with post-hoc Tukey’s test; *P < 0.05, **P < 0.01, ***P < 0.001, n.s., not significant. **G** C-Fos immunofluorescence in the subiculum post-KA or saline induction, illustrating differential activation in ipsilateral vs. contralateral sections. White dashed areas represent the subiculum (Sub.). Scale bar, 50 µm

Although a second dose is not strictly necessary to induce seizures, it does allow more precise temporal control of the seizure onset, which we required. To evaluate the therapeutic potential of the chemogenetic inhibition of subicular pyramidal neurons, we developed an acute kindling model via a second, lower-dose KA injection after eight weeks (Fig. 3A, B). The severity of epileptic seizures was classified according to the Racine grading scale, and mice exhibiting stage 4 or 5 seizures were categorized as having generalized tonic–clonic seizures. Seizure duration was measured for stage 4 or 5 seizures. Epileptic seizures began within 20 min of KA induction. Video footage of mouse seizures in an open field was initially recorded for up to 8 h following injection in three mice; however, we found that seizures occurred intensively within 2 h after acute induction (Fig. 3C, n = 3). Therefore, subsequent experiments all recorded the seizure status of mice for up to 2 h after acute induction in the chronic TLE models. To determine the optimal kindling dose, two doses of KA (1 µg/kg, and 2 µg/kg) were tested via brain cannulation. A significant difference in the kindling rate was observed between the saline (0.9% NaCl) and KA (1 µg/kg, and 2 µg/kg) group (Fig. 3D–F). In the KA group, seizures were stimulated by KA and behavior was observed. As shown in Fig. 3D–F, the total duration of seizures in the 2 µg/kg KA group was higher than that of the saline group, whereas no difference was observed between the 1 µg/kg group and the saline group. Similarly, a significant difference in seizure frequency was only observed with the 2 µg/kg dose (Fig. 3D, E). These results indicate that 2 µg/kg KA is a sufficient dose to induce kindling.

Immunohistochemical analysis confirmed an increase in c-Fos expression within the subiculum following acute KA stimulation with the 2 µg/kg dose. In the saline-induced group, we observed a significantly lower number of c-Fos positive cells than in the KA-induced group (Fig. 3G). These results indicate successful generation of the acute chronic TLE model. This method employs a robust time-controlled protocol to kindle seizures by administration of KA, thus enabling a relatively precise assessment of the chemogenetic silencing effects on epileptic seizures.

### Chemogenetic inhibition of the subiculum alleviates seizure severity in the acute chronic TLE mouse model

**Fig. 4:**
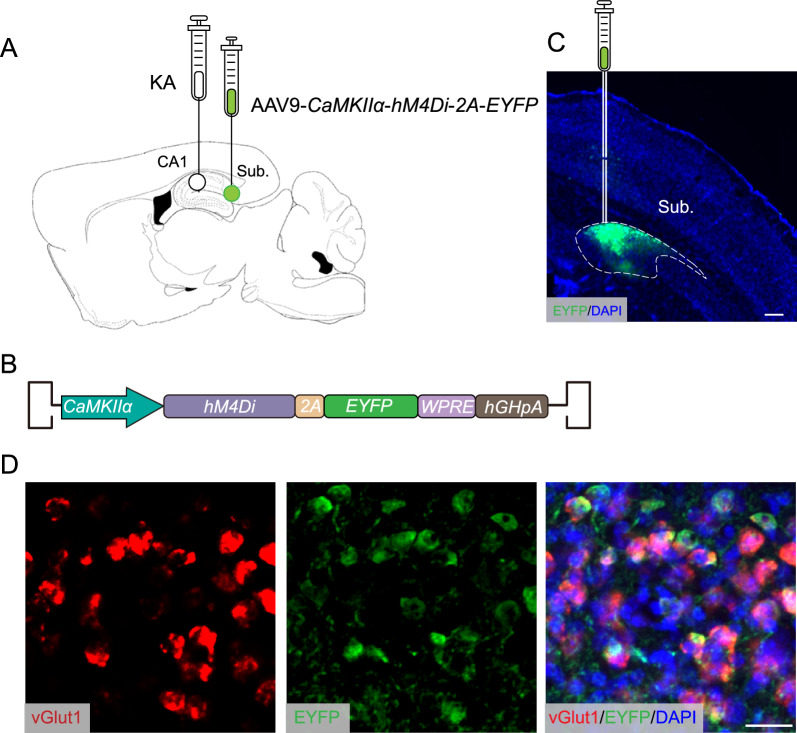
Labeling of excitatory neurons in the subiculum of kainic acid TLE model mice. **A** Schematic showing KA injection sites in the CA1 and AAV9-*CaMKIIα-hM4D*_*i*_*-2A-EYFP* injection sites in the Sub. **B** Schematic showing the AAV9-*CaMKIIα-hM4D*_*i*_*-2A-EYFP* construct. **C** Representative images of the injection site in the Sub. Scale bar, 250 µm. **D** High magnification images of EYFP-positive subicular neurons (green) labeled with *vGlut1* ISH (red), with merged images indicating co-localization (yellow). Scale bar, 20 µm

We next tested the effect of inhibiting subicular pyramidal neurons in KA-kindled chronic TLE mice. We unilaterally injected AAV9-*CaMKII*$\alpha$-*hM4D*_*i*_*-EYFP,* which expresses hM4D_i_ and enables neuronal silencing upon CNO administration, into the subiculum of acute chronic TLE mice (Fig. 4A–C). To confirm reliable hM4D_i_ expression in subicular excitatory pyramidal neurons driven by the CaMKII $\alpha$ promoter, co-localization of EYFP and *vGlut1* cells in brain sections containing the subiculum from these mice was assessed (Fig. 4D). A substantial overlap between these two cell populations was observed. A significant proportion of EYFP-positive cells were positive for *vGlut1*, while a majority of *vGlut1*-positive cells also exhibited EYFP positivity. These results suggest a high degree of specificity and efficiency in the labeling of subicular pyramidal neurons with hM4D_i_-EYFP and indicate that the expression of hM4D_i_ is primarily restricted to excitatory neurons.

**Fig. 5:**
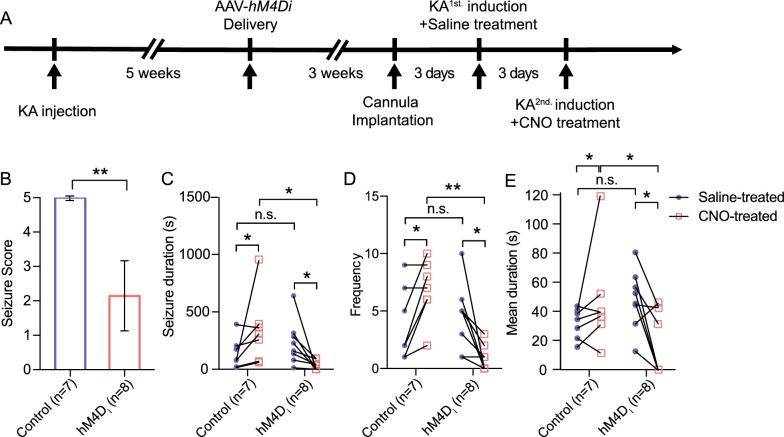
Chemogenetic inhibition of hippocampal subicular excitatory neurons mitigates seizure phenotypes in an acute-chronic TLE model. **A** Schematic showing the timeline of the chemogenetic experiment. **B** Comparison of seizure severity indexed by seizure score between control and hM4D_i_ groups. **C**–**E** Total seizure duration (**C**), seizure frequency (**D**), and mean seizure duration (**E**) for each mouse across both trials. The saline-treated group is denoted by purple dots, and the CNO-treated by red squares. Lines are drawn between the scores of each mouse for comparison (n = 7 control mice, n = 8 h M4D_i_ mice). Data are presented as mean ± SEM, two-tailed paired t-test when comparing two groups between the saline- and CNO-treated groups, and a two-tailed unpaired *t*-test when comparing two groups within the same trial phase; *P < 0.05, **P < 0.01, n.s.: not significant

Next, we tested whether intraperitoneal (i.p.) CNO delivery suppresses generalized tonic–clonic seizures. Following a three-week recovery period for AAV-mediated expression and subsequent cannulation implantation (Fig. 5A), two successive experimental trials were conducted to evaluate KA-induced seizures, each preceded by saline (1st trial) or CNO (2nd trial) treatment.

Prior to chemogenetic inhibition, the potential role of subicular excitatory neurons in modulating seizure manifestations was assessed. As shown in Fig. 5B, under saline treated conditions (7 separate vehicle sessions) all animals displayed score 5 seizures, i.e., rearing with loss of balance. Treatment with CNO significantly suppressed seizure severity (*P* < 0.01). When animals were tested with 1 mg/kg of CNO, the median score was reduced to a 2 (i.e., head nodding). We compared the average duration and frequency of seizures across trials, and noted that, following CNO administration, there was a significant reduction in average seizure duration in the hM4D_i_ group compared to saline-treated group (232.1 $\pm$ 181.1 *vs.* 34.4 $\pm$ 38.7,* P* < 0.05, Fig. 5C), with a concurrent decrease in seizure frequency (4.5 $\pm$ 2.7 *vs.* 0.9 $\pm$ 1.1, *P* < 0.01, Fig. 5D). Moreover, hM4D_i_ mice exhibited a significantly shorter average seizure duration in the second trial following CNO administration than in the first trial following saline (48.9 $\pm$ 19.2 *vs.* 20.6 $\pm$ 21.0, *P* < 0.05, Fig. 5E). Specifically, 7 of 8 mice displayed a reduction in seizure severity after this dose of CNO, and 4 mice were completely protected. However, no significant differences in seizure duration or frequency were observed between the two trials in the control group. Collectively, these results indicate that chemogenetic suppression of hippocampal subicular excitatory neurons alleviate the severity of seizures in a KA-induced acute chronic model of epilepsy.

## Discussion

KA is often used to model epilepsy in rodents, especially refractory TLE. The KA model reliably reproduces the pathological characteristics of TLE, facilitating the exploration of TLE mechanisms and therapeutic interventions [15–17]. However, chronic TLE models induced by local or systemic KA administration are not suited for drug screening due to the unresolved challenge of precisely controlling the timing of generalized seizures. Here, we demonstrate that our TLE model exhibits robust phenotypes, including the rapid onset and high intensity of seizures that occur within 20 min and last for up to 2 h, through a two-step KA administration process. Thus, this model is particularly useful as it offers experimental control over seizure timing, which is ideal for preclinical examination of new approaches to seizure control. This is the first TLE model in which such control over the timing of generalized seizures has been obtained.

The subiculum is important in epileptogenesis, including neuronal synchronization spread from the hippocampus to other regions through subicular projecting pyramidal neurons [6, 9]. In clinical research, it has been shown that TLE patients with secondary generalized seizures (sGS) have reduced volume in the subiculum compared with those without sGS. Consistent with these clinical observations, our finding in the chronic TLE rodent model also revealed reduced volume and elevated apoptosis in the subiculum, implying severe dysfunction in this region.

In addition, neuronal death within the subiculum may play a critical role in influencing epileptogenic circuitry, potentially altering vulnerability to epilepsy and its progression. KA injection has been reported to cause significant structural changes in the subiculum, including the loss of specific neuronal populations, particularly inhibitory interneurons, and upregulation of NPY (Neuropeptide Y) in non-GABAergic neurons, which may contribute to seizure generation and subiculum hyperexcitability [18]. Furthermore, subicular pyramidal neurons may gate the transmission of epileptiform activity from the amygdala through amygdala-CA1-subiculum projections [19]. We have revealed that these inputs to the subiculum from the amygdala, cortex and hippocampus were diminished after chronic TLE. Subicular pyramidal neurons received projections from multiple brain regions, the CA1 area of the hippocampus being the predominant of these. These anatomical observations suggest an important role of subicular pyramidal neurons in seizures originating from the hippocampus. These results also indicate that the subiculum is greatly affected in this chronic TLE model, and that this region may serve as a target for therapeutic intervention. Moreover, an increase in c-Fos expression following acute KA stimulation was observed in the subiculum, suggesting a potential causal relationship between subiculum excitation and generalized tonic–clonic seizure induced by relatively low-dose KA kindling. Consequently, it is evident that there are abnormalities in both the morphology and functional connectivity of the subiculum in the chronic TLE mouse model.

As we demonstrated, the KA acute chronic TLE model was ideal as the first examination of DREADD effects in the subiculum for seizure control. This model not only offers face and construct validity (e.g., it triggers complex-partial seizures, changes in temporal lobe histology, and lasting alterations in excitability) but also allows for tight experimental control over the timing of seizures.

To assess the impact of our targeting on seizure outcomes, 1 mg/kg CNO was administered 30 min before KA kindling. We observed a significant reduction in seizure severity within 2 h of CNO administration lowering seizure scores that typically define status epilepticus. Many patients with refractory epilepsy have seizures that are preceded by premonitory auras, or that occur in predictable clusters (such as in catamenial epilepsy), suggesting potential therapeutic benefits from such chemical-genetic silencing. Using a KA acute chronic TLE model, we demonstrated that targeting subicular excitatory neurons with chemogenetics is an effective tool for suppression of generalized TLE. Previous work has shown that chemogenetic inhibition of the midline thalamus attenuates both the electrographic and behavioral manifestations of amygdala-kindled seizures [12]. Suppression in behavioral seizure severity was seen with doses of CNO as low as 1 mg/kg. Work by Kätzel and colleagues found that chemogenetic silencing of a defined seizure focused on the neocortex (induced by either picrotoxin, pilocarpine, or tetanus toxin in motor cortex of mice) significantly attenuated seizures in each model. They also reported that high doses of CNO (10 mg/kg or 4 mg/kg) had little non-specific CNO-induced effects. In our study, by targeting subicular excitatory neurons in a KA-induced acute chronic TLE model, the administration of 1 mg/kg CNO significantly reduced seizure severity, particularly within two hours of treatment.

In conclusion, this study provides compelling evidence that chemogenetic silencing of subicular pyramidal neurons effectively suppresses generalized tonic–clonic seizures in a mouse model of TLE. By utilizing a precise, two-step kainic acid (KA) administration protocol, we were able to establish an acute-chronic TLE model that offers robust temporal control over seizure onset and duration, ideal for therapeutic testing. The results highlight the critical role of the subiculum in propagating hippocampal seizures, and the success of the DREADD-mediated approach suggests it could be a promising avenue for developing targeted therapies. Future studies will be necessary to explore the long-term effects of this intervention and its potential application in clinical settings for refractory epilepsy treatment.

## Materials and methods

### Animals

We used male C57BL/6J mice (aged 6–20 weeks; Beijing Vital River Laboratory Animal Technology Co., Ltd.) in all experiments. All mice were maintained in our animal house facility on a 12 h light/dark cycle (8:00 A.M. to 8:00 P.M.) and were provided ad libitum access to food and water.

### Recombinant adeno-associated virus (rAAV) vector production

The rAAV utilized in this study was packaged using a triple-plasmid transfection method [20]. Upon reaching 90% confluency, cells were transfected with plasmids required for viral packaging via calcium phosphate transfection. After 72 h in culture, both cells and supernatants were harvested. The enriched cells were subjected to repeated freeze–thaw cycles to lyse the cells, and viral particles were precipitated using PEG8000. For the supernatants, viral particles were collected via high-speed centrifugation. The isolated viruses were resuspended in HEPES-buffered saline (HBS) and preserved at − 80 °C. In this study, rAAV2-retro-*hSyn-EYFP* and AAV9-*CaMKIIα-hM4D*_*i*_*-EYFP* were diluted to achieve a standardized titer of 3 × 10^12^ vector genomes per milliliter (VG/mL). Viral titers were quantified using RT-PCR quantification.

### Stereotaxic surgery for virus injections

For viral injections, mice were anesthetized using sodium pentobarbital (60 mg/kg, i.p.) and positioned in a stereotaxic frame (RWD Life Science, China). Targeted injections were aimed at the subiculum (coordinates, AP: − 3.45 mm, ML: − 2.06 mm, DV: − 1.8 mm) using a volume of 300 nL for virus and 200 nL for AAV retrograde tracing. A small cranial opening was made above the injection site after incision of the scalp. Injections were administered using a 10-µL Hamilton syringe (Hamilton, Switzerland) connected to a microsyringe pump (Legato 130; KD Scientific, Holliston, MA, USA). The needle was retained in place for 10 min post-injection to ensure optimal diffusion. Following the procedure, the incision was sutured with absorbable stitches, and mice were carefully removed from the stereotaxic apparatus. They were then placed on a warming blanket for recovery before being returned to their cages.

### Intrahippocampal kainic acid injection

Mice in the experimental group were anesthetized with pentobarbital at a dose of 75 mg/kg and subsequently positioned on a stereotaxic frame (Reward Life Science, Shenzhen, China). A total of 10 µg/kg of kainic acid (MCE, USA) was precisely infused into the CA1 region of the hippocampus (AP: − 2.06 mm, ML: − 1.35 mm, DV: − 1.60 mm) using a 10-*μ*L Hamilton Neuros syringe, which was seamlessly integrated with an automated injection system (Legato 130). The flow rate was set to a constant 50 nL/min.

### Cannula implantation and intracranial injection

To induce seizures, guide cannulas (0.30-mm diameter, RWD Life Science, China) were surgically implanted directly above the CA1 region of the hippocampus, adhering to specific stereotaxic coordinates (AP: − 2.06 mm, ML: − 1.35 mm, DV: − 1.60 mm). These cannulas were securely anchored to the skull with dental cement to ensure stability. To prevent occlusion, stainless-steel obturators (RSD Life Science, China) were inserted into the guide cannulas and remained in place until the time of infusion.

For the experimental induction of acute epileptic seizures, a total of 2 $\upmu$g/kg of kainic acid was administered into the hippocampal CA1 region via the pre-implanted cannulas in KA-induced group. In contrast, the saline-induced group received an equivalent volume of saline, serving as a negative control for the induction procedure.

### Brain slice preparation

Mice were deeply anaesthetized by i.p. injection of pentobarbital (75 mg/kg) and perfused transcardially with phosphate buffered saline (PBS), followed by 4% paraformaldehyde (4% PFA). Brain samples were postfixed in the 4% paraformaldehyde for 6 h and then placed in 30% sucrose-PBS solution for two days at 4 °C. Next, brains were embedded in Optimal Cutting Compound (OCT) media and frozen on dry ice. Brains was cut into a series of 40-μm thick coronal sections on a cryostat (CM1950; Leica, Wetzlar, Germany).

### Immunohistochemistry

For immunohistochemical analysis, mouse brain sections were first permeabilized and blocked using a solution of PBS containing 0.3% Triton X-100 and 10% bovine serum albumin (BSA, Sigma) for 1 h at room temperature. The sections were then incubated overnight at 4 °C with a goat polyclonal anti-EYFP antibody (1:500; Rockland, CA, USA), followed by a 2-h room temperature incubation with Alexa Fluor 488-conjugated donkey anti-goat IgG (1:500, Thermo Fisher Scientific). For nuclear visualization, sections were stained with 4,6-diamidino-2-phenylindole (DAPI, Sigma, St Louis, MO, USA) for 10 min. After each incubation step, sections were washed thrice with PBS at 10 min/wash. Finally, sections were mounted on slides using an anti-fade aqueous mounting medium (Southern Biotechnology, Birmingham, AL, USA) to preserve fluorescence.

To map TUNEL and EYFP positive cells in Figs. 1E and 2D, 40-μm brain sections covering the entire ROI were placed onto glass slides with 3 sections on each slide. One slide for each mouse that contained matched sections were used for analyses. A Zeiss Axio imager 2 with apotome was used to acquire images for in situ hybridization and immunofluorescence staining. Acquired images were analyzed with Image J.

### In situ hybridization

In situ hybridization was conducted with slight modifications to previously established protocols [11]. Mouse *vGlut1* coding-region sequences were cloned for the synthesis of complementary RNA (cRNA) probes, which were subsequently labeled with digoxigenin (DIG) using the DIG RNA Labeling Mix (Roche, Switzerland). Brain sections were initially treated with 0.3% Triton X-100 for 20 min, followed by a 30-min exposure to 0.3% H_2_O_2_. The sections were then digested with PK protease at 37 °C for 15 min, quenched with 0.75% glycine for 5 min, and acetylated using 0.1 M triethanolamine and 0.25% acetic anhydride for 15 min. Hybridization with DIG-labeled cRNA probes was performed at 56 °C for 13–16 h. Post-hybridization, the sections underwent sequential washes with 2 × SSC at 65 °C for 5 min and two washes with 0.2 × SSC at 65 °C for 20 min/wash. The sections were then incubated with anti-DIG-peroxidase (POD) Fab fragments (1:300, Roche, Switzerland) at 37 °C for 1 h, followed by application of a TSA-plus Cy3 kit (1:100, Perkin Elmer, USA) at room temperature for 30 min in preparation for subsequent immunofluorescence staining.

### Behavioral assays

Prior to experiments, mice were allowed a 5-min adaptation period in an open-field observation chamber (25 × 25 × 25 cm^3^). During the initial trial, mice received an intraperitoneal injection of saline, followed three days later by an administration of Clozapine-N-oxide (CNO, Hello Bio, HB1807; 1 mg/kg). Behavioral evaluations were conducted starting 30 min after the CNO injection for 2 h. Subsequently, seizures were induced by administering 100 nL of kainic acid (KA) through the guide cannulas. Seizure behaviors observed during this period were documented and assigned scores ranging from zero to six, in accordance with the criteria set forth in a modified Racine scale [21, 22].

### Statistical analyses

Statistical analyses and graphical representations were conducted using GraphPad Prism version 8 (GraphPad Software, USA). Data are presented as mean ± SEM. Statistical significance was assessed using two-tailed unpaired Student’s t-test, one-way ANOVA followed by Dunnett’s or Tukey’s post-hoc test. A *P*-value of < 0.05 was considered statistically significant. The specific statistical tests employed for each analysis are detailed in the figure legends.

## Data Availability

The datasets generated and analyzed during the current study are available from the corresponding author upon reasonable request.
